# Mitochondria-acting carrier-free nanoplatform self-assembled by α-tocopheryl succinate carrying cisplatin for combinational tumor therapy

**DOI:** 10.1093/rb/rbab029

**Published:** 2021-06-30

**Authors:** Heng Mei, Jing Li, Shengsheng Cai, Xuequan Zhang, Wenqiang Shi, Hai Zhu, Jun Cao, Bin He

**Affiliations:** 1 National Engineering Research Center for Biomaterials, Sichuan University, No. 29 Wangjiang Road, Chengdu 610064, China; 2 School of Chemical Engineering, Sichuan University, No. 24 South Section 1, Yihuan Road, Chengdu 610065, China

**Keywords:** carrier-free nanodrugs, cisplatin, α-tocopheryl succinate, dual-drug conjugate

## Abstract

Unsatisfactory drug loading capability, potential toxicity of the inert carrier and the limited therapeutic effect of a single chemotherapy drug are all vital inhibitory factors of carrier-assisted drug delivery systems for chemotherapy. To address the above obstacles, a series of carrier-free nanoplatforms self-assembled by dual-drug conjugates was constructed to reinforce chemotherapy against tumors by simultaneously disrupting intratumoral DNA activity and inhibiting mitochondria function. In this nanoplatform, the mitochondria-targeting small-molecular drug, α-tocopheryl succinate (TOS), firstly self-assembled into nanoparticles, which then were used as the carrier to conjugate cisplatin (CDDP). Systematic characterization results showed that this nanoplatform exhibited suitable particle size and a negative surface charge with good stability in physicochemical environments, as well as pH-sensitive drug release and efficient cellular uptake. Due to the combined effects of reactive oxygen species (ROS) generation by TOS and DNA damage by CDDP, the developed nanoplatform could induce mitochondrial dysfunction and elevated cell apoptosis, resulting in highly efficient anti-tumor outcomes *in vitro*. Collectively, the combined design principles adopted for carrier-free nanodrugs construction in this study aimed at targeting different intracellular organelles for facilitating ROS production and DNA disruption can be extended to other carrier-free nanodrugs-dependent therapeutic systems.

## Introduction

Cancer is one of the most deadly diseases threatening the life safety and health for humans, and chemotherapy is currently one of the primary methods of treating tumors [1–3]. Currently, most chemotherapeutic drugs in clinical practice, such as cisplatin (CDDP), paclitaxel (PTX), doxorubicin (DOX), etc., exhibit low bioavailability due to inherent limitations, such as the non-specific distribution, poor bioavailability, rapid blood clearance and poor solubility [4]. In addition, the toxic side effects of chemotherapeutic drugs on normal cells and the production of tumor drug resistance impair their anti-tumor efficacy and further limit their clinical application [5, 6]. Recently, the carrier-assisted drug delivery system (CDDS) has demonstrated promising improvements for treating cancer including enhanced drug solubility and availability, tumor accumulation and penetration and reduced adverse effects [7–9]. However, studies have shown that cancer treatment with a single chemotherapy drug may be insufficient and can lead to drug resistance after several uses [10, 11]. Therefore, combinational therapy by simultaneously delivering multiple drugs has become an effective method to achieve enhanced anti-cancer activity through synergistic/combinational effects. These effects include enhanced tumor suppression efficiency, elevated tumor sensitivity to therapeutic agents, reduced doses of toxic drugs and minimized adverse side effects.

Recently, the burgeoning strategy *via* combining various intracellular-functioning small-molecule drugs has attracted much attention, which could destruct specific subcellular structures and finally result in cell apoptosis. For instance, CDDP, a first-line anti-cancer drug used for the treatment of a variety of solid tumors, can bind with genomic DNA (gDNA) or mitochondrial DNA (mtDNA) to create DNA lesions, activate transduction pathways, or block DNA replication, mRNA transcription and proteins expression, which ultimately leads to cellular necrosis or apoptosis [12–14]. Furthermore, some small-molecular drugs, like curcumin, DOX and PTX, have been co-delivered with CDDP under the assistance of carriers to achieve enhanced therapeutic efficacy [15–18]. Similarly, α-tocopheryl succinate (TOS) as a mitochondria targeting drug with the potential to induce apoptosis in a variety of tumor cells has also aroused great attention [19–21]. Notably, combinational anti-cancer effects were realized when TOS was used in combination with other chemotherapeutic drugs, such as PTX, DOX and Pt^IV^ prodrug [22–25].

Despite significant progress on the ability for CDDSs to deliver dual small-molecular drugs, there are still some obstacles that remain for improving anti-tumor efficacy and clinical conversion. One of the major challenges is that the drug loading capacity is unsatisfactory [e.g. <10% (w/w)] for the majority of drugs, which greatly attenuates the therapeutic effects [26, 27]. In addition, some of the inert materials that only act as vehicles can cause systematic toxicity [28–30]. As a result, carrier-free nanodrugs have been developed to overcome these obstacles. Carrier-free nanodrugs primarily self-assemble through prodrug, pure drug (e.g. single drug or multi drugs) or amphiphilic drug–drug conjugates with minimal use of inert materials, resulting in low systemic toxicity and high drug loading capacity [31–33]. In particular, all the components inside carrier-free nanodrugs composed of multidrugs or amphiphilic drug–drug conjugates can act as the therapeutic agents for combinational therapy.

**Scheme 1.**
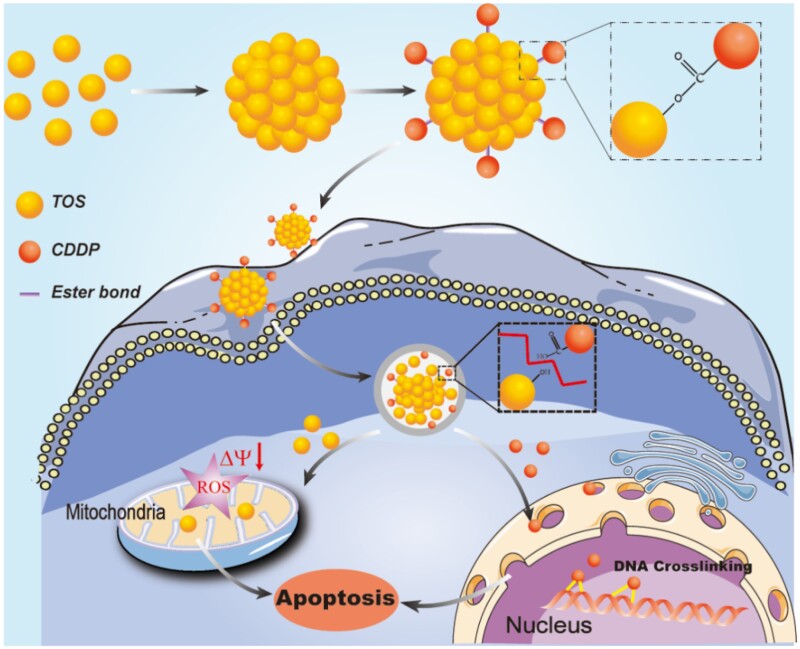
Schematic of the self-assembly processes and the combinational effects of the dual-drug carrier-free nanoplatform.

Herein, we adopted a two-step process to fabricate a carrier-free nanoplatform with dual-drug conjugates to simultaneously damage cellular DNA and mitochondria for combinational cancer therapy (Scheme 1). Firstly, TOS, as the mitochondria targeting small-molecular drug, was self-assembled into carrier-free nanoparticles, followed by the conjugation of CDDP on their surface *via* a pH-responsive linker (i.e. ester bond). In this nanoplatform, TOS not only served as the carrier to deliver CDDP, but also as the mitochondria targeting drug to induce cell apoptosis. Meanwhile, under the combined effects of reactive oxygen species (ROS) generated by TOS and DNA damage caused by CDDP, the dual-drug conjugates carrier-free nanoplatform exhibited excellent anti-tumor effects for suppressing non-small cell lung cancer *in vitro*.

## Materials and method

### Materials

CDDP was purchased from Shanghai yuanye Bio-Technology Co., Ltd. (Shanghai, China). α-tocopheryl succinate (TOS), AgNO_3_ and O-phenylenediamine (OPDA) were obtained from Sigma-Aldrich Co., Ltd. (Steinheim, Germany). Fluorescein isothiocyanate (FITC) was purchased from Sinopharm Chemical Reagent Co., Ltd. Hoechst 33342, 2′,7′-dichlorofluorescein diacetate (DCFH-DA), Annexin V-FITC/PI Apoptosis Detection Kit and Mitochondrial membrane potential assay kit with JC-1 were all purchased from Beyotime Biotechnology Co., Ltd. (Shanghai, China). Roswell Park Memorial Institute (RPMI) 1640 medium, fetal bovine serum and penicillin-streptomycin were ordered from Life Technologies Corporation (Gibco, USA) and directly used for cells tests. Human non-small cell lung cancer cell line (A549) was purchased from the Chinese Academy of Science cells Bank (Shanghai, China).

### Preparation and characterization of CDDP functionalized TOS nanoparticles (CDDP-TOS NPs)

#### The preparation of modified CDDP

The modified CDDP was prepared following the previous studies [34]. In brief, CDDP (10 mg) was dissolved in 9 mL distilled water, and then the silver nitrate ([AgNO_3_]:[CDDP]=1:1, molar ratio) was added to form the aqueous complex and stirred at 37°C overnight in the dark. After that, the mixture was centrifuged at 12 000rpm for 10 min. Prior to collection, the supernatant was filtrated through a 0.22 µm filter.

#### The preparation of TOS NPs

Dropping 240 µL α-TOS solution [25 mg/mL in dimethyl sulfoxide (DMSO)] into 5 mL distilled water while stirring at 37°C for 5 min. Subsequently, the solution was dialyzed overnight to remove DMSO. After that, TOS nanoparticles (TOS NPs) were obtained.

#### The preparation of CDDP-TOS NPs

Firstly, the aqueous solution of the above-prepared CDDP solution (0.56 mL) was added into 5 mL TOS NPs ([CDDP]:[TOS]=1:5, molar ratio), and the pH of the mixture was adjusted to 7.4 by adding NaOH. Then, the neutral mixture was stirring at 37°C for 10 h. After being dialyzed against PBS (pH=7.4) overnight to remove unreacted CDDP, the CDDP-TOS NPs were obtained and stored at 4°C. The preparation method of FITC-labeled CDDO-TOS NPs was similar to that of CDDP-TOS.

The content of CDDP in CDDP-TOS NPs was determined using the OPDA assay [35]. Meanwhile, the content of TOS in CDDP-TOS NPs was measured by HPLC with a C18 column, being eluted by methanol and water (95: 5, v/v) at a flow rate of 1.0 mL min^−1^ at 30°C, and the UV detector set at 288 nm. CDDP-TOS NPs (204.27 μM for CDDP) were lyophilized for ^1^H-NMR (400 MHz, DMSO-*d_6_*, Avance III HD, Bruker Corporation, Germany) and FT-IR (Nicolet 6700, American Thermal Power Company) testing to confirm the structure. The particle size and zeta potential of CDDP-TOS NPs (204.27 μM for CDDP) were measured by dynamic light scattering (DLS) equipment on a Malvern Zetasizer (Nano ZS Malvern, UK). The transmission electron microscope (TEM, Tecnai G2 F20 S-TWIN, American PEI Company) images were captured to observe the morphology of TOS NPs and CDDP-TOS NPs.

### Stability of CDDP-TOS NPs

The stability of CDDP-TOS NPs under different situations, including diluted by deionized water, incubated with serum-rich medium and pH 7.4 buffer solution, was investigated by DLS analysis. Also, the long-term stability of CDDP-TOS NPs was validated by detecting particle size over 7 days.

### pH-sensitive behaviors of CDDP-TOS NPs

To investigate the pH-sensitive behavior of CDDP-TOS NPs, the carrier-free nanoplatform was incubated in the acetic acid–sodium acetate buffer (pH=5.5) at 37°C for 48 h, and incubated in the PBS (pH=7.4) as the control. The sizes of CDDP-TOS NPs after 6, 12, 24 or 48 h incubation were measured through DLS. Moreover, after incubation at pH=5.5 for 48 h, the CDDP-TOS NPs were lyophilized for subsequent Mass Spectrometry (LCMS-IT-TOF, Shimadzu Corporation, Japan) detection.

### Cell culture

A549 cells were cultured in RPMI1640 containing 1% penicillin-streptomycin and 10% FBS and maintained at 37°C incubator with 5% CO_2_ using the standard cell culture procedure.

### Cellular uptake behavior of CDDP-TOS NPs

To investigate the internalization efficiency of the constructed NPs, A549 cells were seeded in glass-bottom plates (*d*=35 mm, 2×10^5^ cells per well) and cultured for 24 h. Then, the culture media were replaced with fresh media containing FITC-labeled CDDP-TOS NPs (95 µM for CDDP) to feed cells for 1, 2 or 4 h with PBS as the control. Subsequently, the nuclei were stained by Hoechst 33342 (10 µg/mL) for 15 min, followed by washing with PBS three times and fixed with 4% paraformaldehyde solution for 20 min at room temperature. Then, confocal laser scanning microscopy (CLSM, TCP SP5, Leica, Germany) was used to observe the fluorescence signal with an excitation wavelength at 488 nm and an emission wavelength at 525 nm.

Flow cytometry (FCM) was used to detect the fluorescence intensity of FITC in A549 cells for quantitatively analyzing CDDP-TOS NPs uptake behavior. In brief, A549 cells were seeded in 6-well plates (*d*=35 mm, 2×10^5^ cells per well) and then treatment procedures were the same as the aforementioned CLSM observation. Afterward, the cells were digested with trypsin, washed three times with PBS, centrifuged, re-suspended in 300 µl of PBS and detected by FCM (LSRfortessa, BD, USA).

### Intracellular ROS generation

DCFH-DA probe was used to detect intracellular ROS generation. A549 cells were seeded in glass-bottom plates (*d*=35 mm, 2×10^5^ cells per well) and cultured for 24 h. Then, the culture medium was replaced with fresh medium containing free CDDP, free TOS and CDDP-TOS NPs (32 µM for CDDP and 160 µM for TOS), respectively, and continued to culture the cells for another 6 h. After that, the culture media were replaced by fresh serum-free media containing DCFH-DA (5 µg/mL), followed by another incubation for 20 min at 37°C. The fluorescence images were immediately acquired *via* CLSM with an excitation wavelength at 488 nm.

For the FCM tests, A549 cells were seeded in 6-well plates (*d*=35 mm, 2×10^5^ cells per well) and the treatment procedures were the same as the aforementioned CLSM observation. Afterward, the cells were digested with trypsin, washed three times with PBS, centrifuged and re-suspended in 300 µL of PBS for analysis. The fluorescence of dichlorofluorescein (DCF) was collected on the FITC channel.

### JC-1 assay

A549 cells were seeded in glass-bottom plates (*d*=35 mm, 2×10^5^ cells per well) and cultured for 24 h. Then, the culture medium was replaced with fresh medium containing free CDDP, free TOS and CDDP-TOS NPs (10 µM for CDDP and 50 µM for TOS), respectively. After 24 h of incubation, the cells were stained according to the provided illustration of mitochondrial membrane potential assay kit with JC-1 (Beyotime Biotechnology, Shanghai, China), and the signals were detected by CLSM with the excitation wavelength at 488 nm for J-monomers and 590 nm for J-aggregates, respectively.

### Cell apoptosis assay

The FCM was used to examine the apoptosis rate of cells. Firstly, A549 cells were seeded in 6-well plates (*d*=35 mm, 2×10^5^ cells per well) and cultured for 24 h. Afterward, the culture media were replaced with fresh media containing free CDDP, free TOS and CDDP-TOS NPs (10 µM for CDDP and 50 µM for TOS), respectively. After 24 h of incubation, the cells were stained according to the provided illustration of Annexin V-FITC/PI Apoptosis Detection Kit (Beyotime Biotechnology, Shanghai, China), and the signals were detected by FCM.

### *In vitro* anti-tumor efficacy

3-[4, 5-dimethylthiazol-2-yl]-2,5-diphe-nyltetrazolium-bromide (MTT) was utilized to measure the cell viability of A549 cells after treatment with different formulations, including free CDDP, free TOS and CDDP-TOS NPs. In brief, A549 cells were inoculated in 96-well plates (1×10^4^ per well) and further cultured for 24 h. Free CDDP, free TOS and CDDP-TOS NPs with varied concentrations (0.125∼56 µM for CDDP and 1 0 ∼200 µM for TOS) were added into each well and incubated with the cells for 24 or 48 h with PBS as the control. Afterward, 100 µL of MTT (0.5 mg/mL) was added to each well and further incubated for another 4 h at 37°C. The supernatant was removed and replaced with DMSO (100 µL). The absorbance of each well at 490 nm was determined by a microplate reader (Model-550, Biorad, America).

### Statistical analysis

Results were expressed as mean±standard deviation (SD). Statistical analysis was performed by using the one-way analysis of variance. *P*<0.05 (*), *P*<0.01 (**) and *P*<0.001 (***) were considered as significant differences.

## Results and discussion

### Preparation and characterization of CDDP-TOS NPs

**Figure 1.**
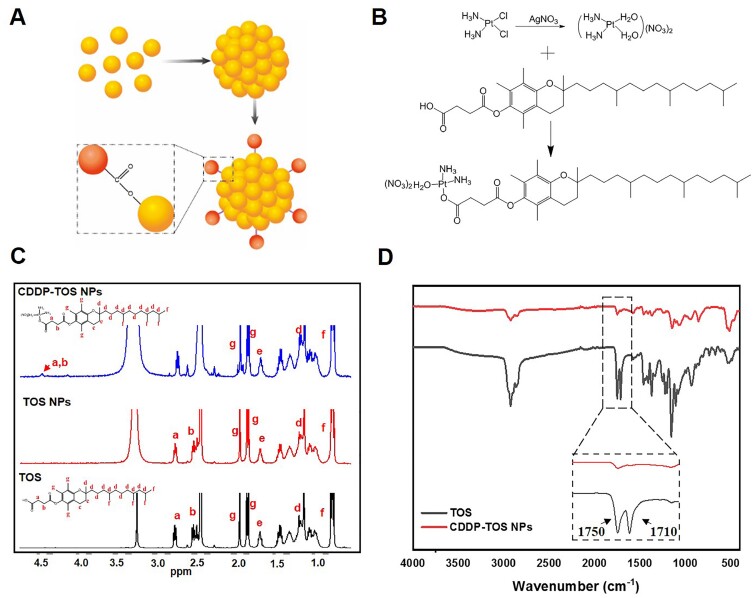
(**A**) The Scheme of the preparation route of CDDP-TOS NPs; (**B**) the synthesis routes of CDDP-TOS NPs; (**C**) the ^1^HNMR spectra of CDDP-TOS NPs, TOS NPs and TOS; and (**D**) the FT-IR spectra of CDDP-TOS NPs and TOS

**Figure 2.**
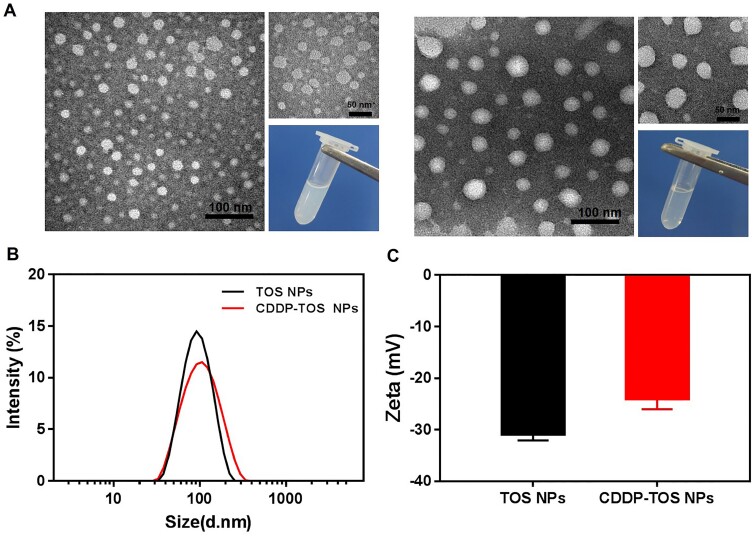
(**A**) The TEM images with different scales (100 and 50 nm) and photos of TOS NPs (left) and CDDP-TOS NPs (right); the size (**B**) and zeta potential (**C**) of TOS NPs (1.029 mM) and CDDP-TOS NPs (204.27 μM for CDDP). All data are expressed as mean±SD (*n*=3).

The fabrication of CDDP-TOS NPs was different from the carrier-free nanodrugs that self-assembled by two various drugs or dual-drug conjugates [36, 37]. Specifically, the synthesis process of CDDP-TOS NPs was categorized into two steps involving the preparation of TOS NPs and subsequent conjugation of modified CDDP on TOS NPs’ surface, as depicted in Fig. 1A. The uniformly dispersed TOS NPs were constructed *via* a universal dialysis method based on the mitochondria targeting small-molecular drug, TOS. It is observed that TOS could be easily self-assembled into a well-defined spherical morphology (Fig. 2A). Notably, the ^1^HNMR spectra results (Fig. 1C) validated the similar structures of TOS molecules and TOS NPs, confirming that the synthesis of TOS NPs did not change the inherent structure or drug activity of TOS. Moreover, the constructed TOS NPs exhibited a negative charge (−31 mV), attributing to the abundant functional carboxyl groups in the TOS molecules, which were expected to be easily tethered with the oxhydryl of aquated CDDP onto their surface *via* a classic esterification reaction [38]. The slightly increased particle size and zeta potential both clearly indicated the formation of CDDP-TOS NPs after conjugation with CDDP, which was further demonstrated by TEM results showing larger spherical nanoscale particles (Fig. 2A).

The conjugation procedure was shown in Fig. 1B, and ^1^HNMR and FT-IR were performed to verify the successful conjugation. As shown in Fig. 1C, a typical new peak at δ=4.52 ppm (a–b) attributed to methylene next to the ester bond in CDDP-TOS NPs appeared, while other peaks were all in their corresponding sites, demonstrating the successful synthesis of CDDP-TOS NPs. Additionally, the changes of signal intensity for the ν(C=O) (1710 cm^−1^ and 1750 cm^−1^) signals of carboxyl in the FT-IR spectra for both TOS and CDDP-TOS NPs indicated the successful synthesis of CDDP-TOS NPs. Furthermore, no other differences were observed in the spectra, which indicated no effects on the structure of TOS after modification. The concentration of CDDP and TOS in the CDDP-TOS NPs, measured by UV and HPLC, were 204.27 μM and 1.029 mM, respectively, calculated in reference to the standard solutions of CDDP and TOS (Supplementary Fig. S1).

### Stability evaluation of CDDP-TOS NPs

**Figure 3.**
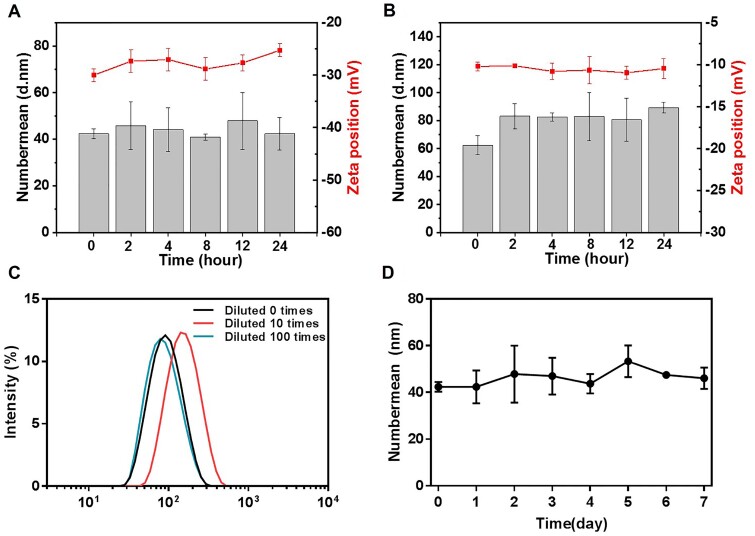
The size and zeta potential changes of CDDP-TOS NPs in PBS (**A**) and 10% FBS (**B**); (**C**) the size changes of CDDP-TOS NPs under different dilution times; and (**D**) the size change of CDDP-TOS NPs in 7 days. All data are expressed as mean±SD (*n*=3).

The negatively charged surface potential of the constructed nanoplatform is generally beneficial for achieving high stability associated with extended blood circulation time. In this study, DLS was carried out to monitor the hydrodynamic diameter changes of CDDP-TOS NPs in different mimic environments. As we expected, the incubation of CDDP-TOS NPs in PBS or serum-riched medium did not change their particle sizes and zeta potential (Fig. 3A and B), indicating good stability in both biomimetic environments. Alternatively, the particle sizes of CDDP-TOS NPs varied little after a 100 times dilution as shown in Fig. 3C. As we know, during the *in vivo* journey, NPs will not only face challenges, such as serum-protein adsorption, but also may be affected by the disintegration after being diluted. Therefore, the encouraging *in vitro* results, i.e. stable particle size and negative zeta potential, have shown promising results for stable particles *in vivo* and subsequent delivery to the target site for cancer cells apoptosis. Intriguingly, the particle sizes after being stored at 4°C for 7 days were also consistent (Fig. 3D), further suggesting their excellent stability and long-term storage potential. Collectively, the constructed dual-functional carrier-free nanodrugs were endowed with prolonged blood circulation and could maintain their original physiochemical properties under low concentrations and long-term storage conditions.

### pH-sensitive evaluations

**Figure 4.**
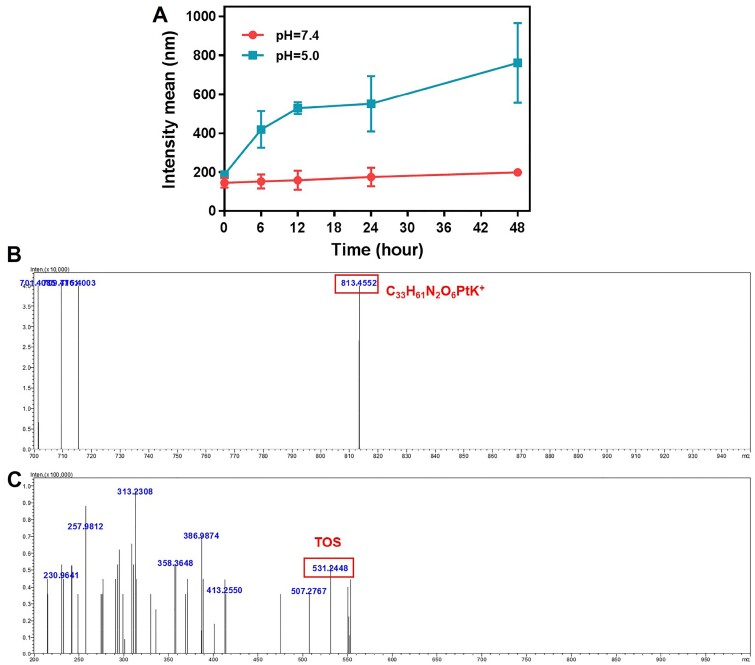
(**A**) The size changes curves of CDDP-TOS NPs in different pH conditions; the MS spectra (ESI+) of CDDP-TOS NPs (**B**), and CDDP-TOS NPs after incubating at pH=5.5 conditions for 48 h (**C**). All data are expressed as mean±SD (*n*=3).

The pH-responsive property of the CDDP-TOS NPs is crucial for successful intracellular release of CDDP. To verify this behavior, we stimulated the pH-induced particle size change *in vitro*. Firstly, we incubated CDDP-TOS NPs in pH=7.4 or 5.5 buffer solution for 48 h to investigate the size difference of the CDDP-TOS NPs. As shown in Fig. 4A, under the pH=7.4 condition, the size of CDDP-TOS NPs almost had no change. In contrast, the size of CDDP-TOS NPs became larger with prolonged incubation time under the acidic condition (pH=5.5). The larger particle size could be attributed to the breakage of the ester bond between CDDP and TOS and further influenced on the structure of TOS NPs. Furthermore, the mass spectrometry (MS) directly verified the breakage of the linker in the CDDP-TOS NPs. According to the MS results, the m/z of CDDP-TOS was observed at 813.4552, which was consistent with the calculated value (814.37, C_33_H_61_N_2_O_6_PtK^+^). After incubating at pH=5.5 at 37°C for 48 h, the m/z changed to 531.2448, where the calculated value of TOS was 530.79 and TOS Na^+^ was 553.78. The decreased MS after incubation with the acidic solution (pH=5.5) indicated the CDDP could be successfully released from CDDP-TOS NPs. In all, the results above strongly proved that CDDP-TOS NPs have the ability to exhibit intracellular drug release, facilitating the drugs aimed at the target sites to exert their functions.

### Intracellular internalization efficacy

**Figure 5.**
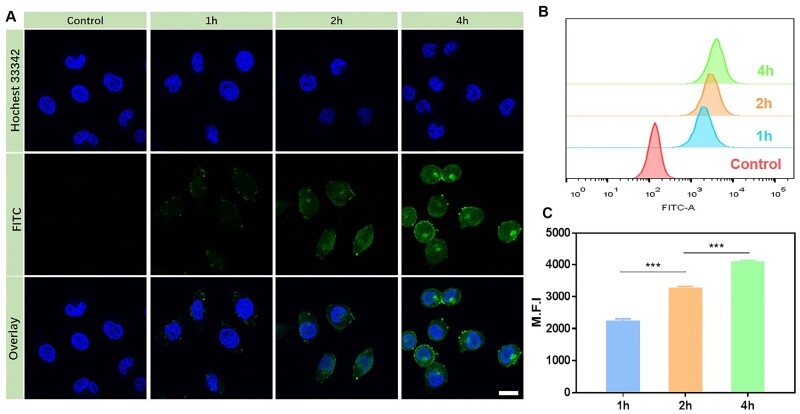
CLSM images (**A**), FCM results (**B**) and quantitative fluorescence intensity (**C**) of A549 cells after FITC-labeled CDDP-TOS NPs treatment for 1, 2 and 4 h. All data are expressed as mean±SD (*n*=3). Scale bar: 25 μm.

Intracellular uptake is a prerequisite for tumor therapy, which facilitates the delivery of therapeutic agents to specific sites. To evaluate the internalization behavior of CDDP-TOS NPs, FITC-labeled CDDP-TOS NPs with similar particle size and surface charge were successfully fabricated (Supplementary Fig. S2). Herein, the time-dependent uptake of CDDP-TOS NPs by A549 cells was initially evaluated by CLSM and FCM. The green fluorescence representing FITC-labeled CDDP-TOS NPs was clearly observed in A549 cancer cells after 1 h incubation and gradually increased as the incubation time was prolonged (Fig. 5A), suggesting increasing CDDP-TOS NPs accumulation in A549 cancer cells. Surprisingly, when the incubation time increased to 4 h, some green fluorescence was detected in the nucleus, indicating the NP’s ability to deliver CDDP into specific intracellular sites. As we know, CDDP induces cell apoptosis through crosslinking with the purine bases on the DNA to interfere with DNA repair mechanisms. Thus, the constructed carrier-free nanoplatform presented the great potential to improve their anti-tumor efficacy through intervening with the DNA. In addition, FCM results also suggested more accumulation of CDDP-TOS NPs in A549 cancer cells with increasing incubation time (Fig. 5B). In particular, when the incubation time was 4 h, the fluorescence intensity in A549 cancer cells was 1.2-fold and 1.8-fold higher than that of an incubation time of 2 and 1 h, respectively (Fig. 5C). These results demonstrate the prominent internalization behavior of CDDP-TOS NPs as a key role in elevating the inhibitory outcome against cancer cells.

### JC-1 assay

**Figure 6.**
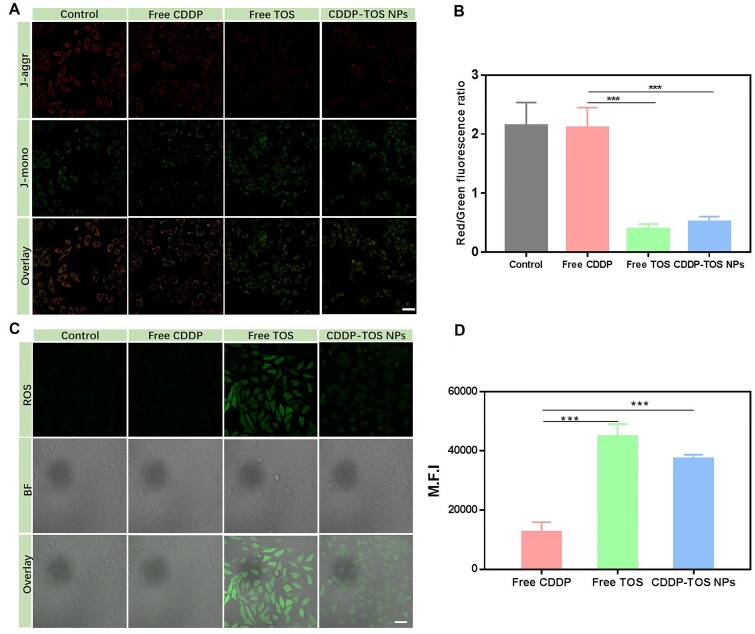
CLSM images (**A**) and semi-quantitative red/green fluorescence ratio (**B**) of A549 cells stained by JC-1 after different treatments for evaluating mitochondrial membrane depolarization; CLSM images (**C**) and quantitative ROS fluorescence intensity (**D**) of A549 cells stained by DCFH-DA after different treatments for evaluating ROS production level. All data are expressed as mean±SD (*n*=3). Scale bar: 75 μm.

After the constructed nanoplatform internalized into cells, we examined the effects of CDDP-TOS NPs on mitochondrial function because TOS as the mitochondria-targeted drug can selectively destabilize the mitochondria function of cancer cells. The mitochondrial membrane depolarization is a specific sign of apoptosis, which mainly occurs in the early stage of apoptosis [39, 40]. JC-1 is an ideal fluorescent probe widely used in mitochondrial membrane potential detection, which can determine the mitochondrial membrane potential changes in cell, tissue or purified mitochondrial membrane. When the mitochondrial membrane potential is high, JC-1 aggregates in the matrix of the mitochondria to form a polymer, which can produce red fluorescence. When the mitochondrial membrane potential is low, JC-1 exists in the form of a monomer, and can produce green fluorescence [41]. This change in fluorescence color signals the change in mitochondrial membrane potential, and the relative ratio of red to green fluorescence is also a measurement of the ratio of mitochondrial depolarization. In this study, the mitochondria membrane potential (ΔΨm) was visualized by CLSM using the intracellular fluorescence intensity of JC-1. As shown in Fig. 6A, when the cells were treated with free CDDP, which has no impact on mitochondria membrane potential, the red fluorescence was stronger than green fluorescence, which was comparable to the control group. However, the red fluorescence became greatly reduced, accompanied by a stronger green fluorescence in cells after being treated with free TOS or CDDP-TOS NPs. The reduction of red fluorescence intensity in A549 cells with TOS and CDDP-TOS NPs treatment represents the depletion of mitochondrial transmembrane potential. We also semi-quantified the red and green fluorescence from CLSM results and compared the ratio of red to green fluorescence in A549 cells with different treatments (Fig. 6B). In agreement with CLSM results, the red/green fluorescence ratio in the cells treated with free TOS or CDDP-TOS NPs decreased significantly. Notably, the ratio of red to green fluorescence in cells treated with free TOS was slightly smaller than cells treated with CDDP-TOS NPs, which could be because the small molecules entered into cells are more easily than nanoparticles.

### Intracellular ROS generation detection

As validated above, the constructed nanoplatform can disturb the mitochondria membrane, which means the CDDP-TOS NPs are distributed in the mitochondria. It is well known that TOS, as a complex II inhibitor in the electron transport chain, could replace the coenzyme Q that binds to the mitochondrial inner membrane complex II, after entering the mitochondria, causing the production of ROS [42]. A fluorescence probe, DCFH-DA, was used to monitor ROS production. Initially, the fluorescence probe could freely pass through the cell membrane and exhibits no fluorescence. After entering the cell, DCFH-DA can be hydrolyzed by intracellular esterase to produce DCFH. Intracellular ROS can oxidize non-fluorescent DCFH to produce green fluorescent DCF, resulting in the intensity of green fluorescence to be directly proportional to the level of ROS [43, 44]. To further verify whether the CDDP-TOS NPs internalized into cells can produce ROS in A549 cancer cells, the intracellular ROS production was qualitatively evaluated by a DCFH-DA probe under CLSM and quantitatively measured by FCM. Figure 6C shows that the green fluorescence representing ROS was clearly observed in free TOS and CDDP-TOS NPs groups, whereas no obvious green fluorescence was detected in the free CDDP group and was comparable to the control group. These results demonstrated that TOS showed a strong capability to generate ROS and tethered CDDP on the surface of TOS NPs did not modify the functionality of free TOS. According to the FCM results, the fluorescence intensity in cells treated with CDDP-TOS NPs was about 3-fold higher than cells treated with free CDDP, but slightly lower than cells treated with free TOS (Fig. 6D). This can be attributed to the different internalization pathways of free TOS and CDDP-TOS NPs demonstrated by other studies [45]. In general, TOS can be more easily diffused into cells *via* passive diffusion, while the CDDP-TOS NPs are mainly internalized into the cells *via* the endocytosis pathway and released from the nanoparticles before exerting the anti-tumor activity, resulting in a reduced amount of TOS located in cells and ROS generation. Overall, the ability for the constructed CDDP-TOS NPs to generate ROS demonstrated their potential to inhibit cancer cells through destroying the mitochondria function, which in combination with CDDP can further improve the anti-tumor efficiency though the destruction of specific subcellular structures.

### *In vitro* cytotoxicity evaluation

**Figure 7.**
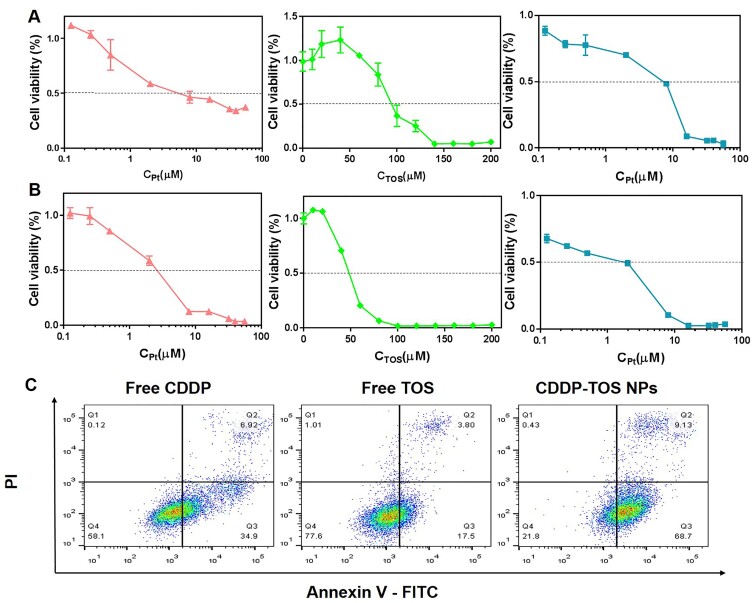
The cell viability of A549 cells upon treatment with free CDDP (red), free TOS (green) and CDDP-TOS NPs (blue) for 24 h (**A**) or 48 h (**B**). All data are expressed as mean±SD (*n*=5). (**C**) FCM patterns of A549 cells after different treatments for quantifying cell apoptosis. All data are expressed as mean±SD (*n*=3).

Enlighted by the ROS production from TOS and DNA damage from CDDP, the combined anti-tumor behavior using two different anti-tumor mechanism drugs was evaluated and detected by MTT assay, and the 50% inhibiting concentration (IC_50_) for different treatments with A549 cells after 24 and 48 h incubations were summarized in Table 1. After being incubated with CDDP-TOS NPs of varying concentrations for 24 h, the IC_50_ of CDDP-TOS NPs was 7.3 µM, while the IC_50_ of free CDDP and free TOS were 5.3 µM and 95 µM, respectively (Fig. 7A). The lower IC_50_ value of free CDDP can be attributed to the rapid entrance of small-molecular CDDP, while the nanoparticles need more time to be internalized. When the cells were incubated with free CDDP or free TOS for 48 h, the IC_50_ decreased to 2.6 µM and 48.4 µM, respectively, while the IC_50_ of CDDP-TOS NPs greatly decreased to 2.0 µM (Fig. 7B), suggesting more free molecules and NPs were accumulated at the tumor cells and exerted their anti-tumor activities. Intriguingly, better anti-tumor efficacy was observed in CDDP-TOS NPs compared to the free CDDP and free TOS molecules, further demonstrating that the combination of TOS and CDDP together to fabricate NPs contributed to enhanced the anti-tumor efficacy.

**Table 1.** The IC_50_ values of free CDDP, free TOS and CDDP-TOS NPs for 24 or 48 h

	Free CDDP	Free TOS	CDDP-TOS NPs
IC_50_ of 24 h (μM)	5.3	95.0	7.3
IC_50_ of 48 h (μM)	2.6	48.4	2.0

### Cell apoptosis assay

In order to determine the possible mechanism for the cytotoxicity that was induced by the combination of TOS and CDDP, the apoptosis of A549 cells after treatments with different groups was investigated by Annexin V-FITC and PI double-staining assay and FCM assay. Due to massive ROS production from TOS and replication disruption caused by CDDP binding to DNA, A549 cells treated with CDDP-TOS NPs for 24 h resulted in 68.7% and 9.13% early and late apoptosis, respectively (Fig. 7C). It was also found that free CDDP induced 34.9% and 6.92% cells in early and late apoptosis, and free TOS induced 17.5% and 3.8% cells in early and late apoptosis with the same treatment. Since 50 µM is much lower than the IC_50_ of the free TOS (95 µM), it resulted in less cell apoptosis. Furthermore, despite 10 µM being higher than the IC_50_ of free CDDP and CDDP-TOS NPs, the drug resistance of free CDDP at high concentrations may rendered the apoptosis of A549 cells treated with CDDP less effective [14, 46], whereas drug resistance for CDDP-TOS NPs did not appear. The above results indicated that the combination of ROS generated by TOS and the damage of DNA by CDDP in a constructed nanoplatform could further improve the anti-tumor performance and ultimately achieve excellent anti-tumor outcomes.

## Conclusion

In summary, a combined strategy of destroying different intracellular organelles has been established and equipped to CDDP-TOS NPs for potentiating chemotherapy against non-small cell lung cancer. This dual-drug conjugated carrier-free nanoplatform with spherical nanoscale particle sizes and a negative surface charge was shown to efficiently internalize into tumor cells, destroy mitochondria function through ROS generation by TOS, and augment the tumor apoptosis ability combined with CDDP *via* binding with gDNA or mtDNA to create DNA lesions. Due to combinational effects through the generation of ROS and DNA disruption, the CDDP-TOS NPs showed improved anti-tumor efficacy compared to free drugs and exhibited an excellent combinational suppressing tumor growth effect. We believed that our strategy is highly valuable for developing dual-drug carrier-free nanoplatform systems to achieve enhanced therapeutic efficacy.

## Supplementary data

Supplementary data are available at *REGBIO* online.

## Supplementary Material

rbab029_Supplementary_Data
