# Supplementary material for: Mitochondria-acting carrier-free nanoplatform self-assembled by α-tocopheryl succinate carrying cisplatin for combinational tumor therapy
Source: Regen Biomater. 2021 Jun 30;8(4):rbab029. doi: 10.1093/rb/rbab029 (PMC8242230; doi:10.1093/rb/rbab029)
Supplement: rbab029_Supplementary_Data [file rbab029_supplementary_data.zip › Supporting Information with Changes Marked.doc]

***Supporting Information***

**Mitochondrial dysfunctional carrier-free nanoplatform self-assembled by α-tocopheryl succinate carrying cisplatin for combinational tumor therapy**

*Heng Mei ^a^, Jing Li ^b^, Shengsheng Cai ^a^, Xuequan Zhang ^a^, Wenqiang Shi ^a^, Hai Zhu ^a^, Jun Cao ^a,^*, Bin He ^a^*

*^a^ National Engineering Research Center for Biomaterials, Sichuan University,*

*Chengdu 610064, China.*

*^b^ School of Chemical Engineering, Sichuan University, Chengdu 610065, China*

*Corresponding author. Tel.: 86-28-85470770; Fax: 86-28-85410246

E-mail address: caojun@scu.edu.cn

**Supplementary figures**

**Figure S1.** The standard curves of CDDP (A) and TOS (B).

**Figure S2.** The size (A) and zeta potential (B) of FITC-labeled CDDP-TOS NPs.
