# Supplementary material for: Mitochondria-acting carrier-free nanoplatform self-assembled by α-tocopheryl succinate carrying cisplatin for combinational tumor therapy
Source: Regen Biomater. 2021 Jun 30;8(4):rbab029. doi: 10.1093/rb/rbab029 (PMC8242230; doi:10.1093/rb/rbab029)
Supplement: rbab029_Supplementary_Data [file rbab029_supplementary_data.zip › Response to reviewers.docx]

**Dear Editor,**

Many thanks for your work and giving us the opportunity to revise our manuscript (RB-2021-065). We would like to thank the reviewers for their constructive comments, which have helped us improve the quality of the paper. We have revised the manuscript carefully in accordance to their suggestions. All the changes have been highlighted in yellow in the revised manuscript. Please find attached our responses to the comments of reviewers.

Please let us know if you have any further questions. Thank you again for your consideration.

Yours sincerely,

Prof. Jun Cao ([caojun@scu.edu.cn](mailto:caojun@scu.edu.cn))

National Engineering Research Center for Biomaterials Sichuan University, Chengdu, 610064, China

**Comments from reviewer 1:**

*In this work, the authors present an interesting nanoparticle system for the delivery of cisplatin as an anticancer therapy. The authors present this as a "carrier free" system, which is taken to imply that the nanoparticles do not contain inert polymer or lipid materials, but rather are composed of agents with their own pharmacological activity. While not a new idea, this is a very nice implementation of the concept. However, there are some questions regarding the NP composition and how data is presented that limit the understanding of what is reported.*

***Response***: Thanks for your positive comments and kind recommendation.

*1.* *What is the heterogeneity of the particles? How uniformly distributed is the Cisplatin coupling? Importantly, how many copies of cisplatin per particle or on a per TOS ratio is there?*

***Response:*** Thanks for the reviewer’s constructive suggestion, which are helpful for improving our manuscript. The low size dispersity and homogeneous spherical morphologies (Figure 2A, B in the manuscript) stated that the fabricated CDDP-TOS NPs have good uniformity. In order to investigate the distribution of CDDP (Pt) on the surface of CDDP-TOS NPs, elemental mapping was performed. As shown in Figure 1, the red dots, which represent Pt, were evenly distributed throughout the nanoparticle, indicating the uniform distribution of CDDP on the nanoparticles. Moreover, the contents of CDDP and TOS in CDDP-TOS NPs were measured by UV-vis and HPLC respectively. According to the results, there were 204.3 μM CDDP and 1.029 mM TOS in CDDP-TOS NPs. The calculated ratio of CDDP to TOS in CDDP-TOS NPs is 1:5, which is consistent with the feed ratio.

Figure 1. (A-B) the TEM images of CDDP-TOS NPs with different scales. (C) The elemental mapping image of Pt. (D) Merged images of (B-C).

*2. These questions are important as they inform what is the actual action being observed. For instance, the TOS particles show higher incidence of ROS generation. it is known that tocopheryls can suppress or induce ROS in a dose dependant manner. What is the relative concentration in the tocopheryls (modified/unmodified) in the two groups presented? Does that explain the reduced ROS with cisplatin or is cisplatin directly inhibiting ROS generation?*

***Response:*** We are sorry to make this point confusing. As shown in Figure 2, it is free TOS rather than TOS particles could induce higher level of ROS generation, which may be attributed to the different internalization pathways between free TOS and TOS nanoparticle form. Free drugs enter the cellular cytoplasm *via* rapid passive diffusion across the cellular membrane and accumulate quickly in cells to play antitumor activities. In contrast, nanoparticles enter into tumor cells *via* endocytosis, which is a slower and less-effective process, compared to passive diffusion [1]. Thus, more TOS could be accumulated in the cells treated with free TOS, resulting in higher level of ROS generation compared to the group treated with TOS particles. Moreover, the intracellular ROS level treated with TOS particles is also lower than both TOS NPs + free CDDP and CDDP-TOS NPs groups, indicating the combined contribution of TOS and CDDP for ROS generation. Additionally, the relative concentration of α-tocopheryls in all groups (i.e., free TOS, TOS NPs, TOS NPs+free CDDP and CDDP-TOS NPs) presented are the same (160 µM). The calculation is as following. Briefly, as measured by UV-vis and HPLC, the concentration of TOS in CDDP-TOS NPs was five times that of CDDP, and we chose the concentration of CDDP in CDDP-TOS NPs to represent the concentration of NPs. In the intracellular ROS generation study, the concentration of CDDP-TOS NPs (TOS modified) was 32 µM ([CDDP] = 32 µM, [TOS]=160 µM), so the concentration of free TOS (not modified and not particles) used in ROS generation study was 160 µM.

Figure 2. The CLSM images of A549 cells with different treatments (A), and the semi-quantitative results (B). scale bar: 75 μm.

*3. What is the meaning of concentration in the TOS-cisplatin group in the IC_50_ studies? Is it the concentration of cisplatin? of tocopheryl or the particle conjugates? Is the observed IC50 synergistic or simply additive effects?*

***Response:*** Thanks for your constructive suggestion. The concentration of CDDP-TOS NPs group in the IC_50_ studies is the concentration of CDDP in the nanoparticles.

We use the Chou-Talalay method to determine whether the drug combination effect was synergistic, additive or antagonistic [2]. Combination index (CI) values are calculated by the following formula:

$$CI=\frac{D_{1}}{{(Dx)}_{1}}+\frac{D_{2}}{{(Dx)}_{2}}$$

where (Dx)_1_ and (Dx)_2_ represent the IC_50_ value when drug 1 or 2 acts singly. D_1_ and D_2_ represent the concentrations of drug 1 and drug 2 when given as a pair at the IC_50_ value. Using this method, CI>1 indicates antagonism, CI=1 indicates additive, while CI<1 indicates synergism.

The CI value of CDDP-TOS NPs for 48 hours’ incubation is 0.97, which indicating that CDDP-TOS NPs could induce additive effects by combining both drugs into a single nanoparticle.

*4.* *Is there a theoretical limit on particle stability associated with cisplatin coupling?*

***Response:*** Thanks for your constructive question. In order to find the limitation of the particle stability associated with CDDP coupling, we synthesized CDDP-TOS NPs with different feed ratio ([CDDP] : [TOS]=1:5, 1:4, 1:2, 1:1, 2:1, molar ratio). Firstly, we measured the size and zeta potential by DLS, and the results were shown in Table 1. According to the results, when the ratios were 1:5, 1:4, and 1:2, uniform nanoparticles were obtained, but it failed to get nanoparticles with appropriate particle size at 1:1 and 2:1. We further measure the stability of the nanoparticles of three ratios. As shown in Figure 3, all the nanoparticles had good stability in PBS for incubating at room temperature for 24 h. So, 1:2 is the limit ratio on particle stability.

Figure 3. The size (A) and zeta potential (B) of CDDP-TOS NPs with different ratios.

Table 1. The size, PdI, zeta potential and concentration of CDDP-TOS NPs with different ratios.

| [CDDP]:[TOS] | Size (nm) | PdI | Count Rate | Attenuator | Zeta potential (mV) |
| --- | --- | --- | --- | --- | --- |
| 1:5 | 174.2 | 0.223 | 314.1 | 8 | -29.9 |
| 1:4 | 169.2 | 0.260 | 335.7 | 8 | -30.8 |
| 1:2 | 158.9 | 0.227 | 396.5 | 8 | -25.8 |
| 1:1 | -- | 0.426 | 121.7 | 11 | -18.8 |
| 2:1 | -- | 0.919 | 184.9 | 11 | -16.9 |

**Comments from reviewer 2:**

*Mei et al., developed a carrier-free nanoplatform for the delivery of cisplatin. The nanoparticles were composed of self-assembled a-tocopheryl (small molecule which targets the mitochondria) in which cisplatin was conjugated to. The performed experiments demonstrated synthesis of negatively charged nanoparticles which are physiologically stable, pH-responsive, and taken up by A549 cells. The delivered cargo to the cells resulted in generation of ROS, mitochondrial dysfunction, and increased cell apoptosis.*

*After review of this manuscript, I recommend acceptance upon revision.*

*To strengthen this manuscript, I advise this paper to be re-read and revised by native English speakers for sentence structure and general flow. Please refer to the points below for specific comments/revisions.*

***Response***: Thanks for your positive comments and kind recommendation.

1. *First sentence of the abstract needs revision for clarification to link to the second sentence regarding “above concerns”*

***Response:*** Thanks for your constructive suggestion. We have revised the first sentence of the abstract to make it more reasonable.

Following are the revisions:

“Unsatisfactory drug loading capability, potential toxicity of the inert carrier, and the limited therapeutic effect of a single chemotherapy drug are all vital inhibitory factors of carrier-assisted drug delivery systems for chemotherapy. To address the above obstacles, a series of carrier-free nanoplatforms self-assembled by dual-drug conjugates was constructed to reinforce chemotherapy against tumors by simultaneously disrupting intratumoral DNA activity and inhibiting mitochondria function.”

2. *Please revise the abstract for general flow*

***Response:*** Thanks for your advice. We have revised the abstract.

Following are the revisions:

“Unsatisfactory drug loading capability, potential toxicity of the inert carrier, and the limited therapeutic effect of a single chemotherapy drug are all vital inhibitory factors of carrier-assisted drug delivery systems for chemotherapy. To address the above obstacles, a series of carrier-free nanoplatforms self-assembled by dual-drug conjugates was constructed to reinforce chemotherapy against tumors by simultaneously disrupting intratumoral DNA activity and inhibiting mitochondria function. In this nanoplatform, the mitochondria-targeting small molecular drug, α-tocopheryl succinate (TOS), firstly self-assembled into nanoparticles, which then were used as the carrier to conjugate cisplatin (CDDP). Systematic characterization results showed that this nanoplatform exhibited suitable particle size and a negative surface charge with good stability in physicochemical environments, as well as pH-sensitive drug release and efficient cellular uptake. Due to the combined effects of reactive oxygen species (ROS) generation by TOS and DNA damage by CDDP, the developed nanoplatform could induce mitochondrial dysfunction and elevated cell apoptosis, resulting in highly efficient anti-tumor outcomes *in vitro*. Collectively, the combined design principles adopted for carrier-free nanodrugs construction in this study aimed at targeting different intracellular organelles for facilitating ROS production and DNA disruption can be extended to other carrier-free nanodrugs-dependent therapeutic systems.”

3. *Focus on sentence transitions throughout the manuscript*

***Response:*** Thanks for your suggestion. We have strengthened the transition of sentences in the manuscript, such as line 3, line 6 and line 9 in Section 1. All changes have been highlighted in the manuscript.

4. *Revise sentence structure of introduction to strengthen manuscript*

***Response:*** Thanks for your constructive suggestion. We have revised the sentence structure of the introduction, and all changes have been highlighted in the manuscript.

5. *Double check that all acronyms are defined the first time*

***Response:*** Thanks for your constructive suggestion. We checked all acronyms in the whole manuscript and completed the full names of some acronyms, such as ROS, DCF, FCM, and MTT.

Following are the revisions:

- Due to the combined effects of reactive oxygen species (ROS) generation by TOS and DNA damage by CDDP……
- The fluorescence of dichlorofluorescein (DCF) was collected on the FITC channel.
- Flow cytometry (FCM) was used to detect the fluorescence intensity of FITC in A549 cells……
- 3-[4, 5-dimethylthiazol-2-yl]-2, 5-diphe-nyltetrazolium-bromide (MTT) was utilized to measure the cell viability of A549 cells……

6. *Edit methods section, so that sentences do not start with numbers (ex. Section 2.2 line 29 and line 49)*

***Response: Thanks for your constructive suggestion.*** We have revised all sentences started with numbers, including Section 2.2 line 7 and line 17.

Following are the revisions:

“*The preparation of TOS NPs:* Dropping 240 μL α-TOS solution (25 mg/mL in dimethyl sulfoxide) into 5 mL distilled water while stirring at 37 ^o^C for 5 minutes. …… The preparation method of FITC-labeled CDDO-TOS NPs was similar to that of CDDP-TOS.”

7. *Include the solvent used for conducting H NMR and the concentration of nanoparticles tested*

***Response:*** Thanks for your advice. We have supplemented the solvent used for conducting ^1^HNMR and the concentration of nanoparticles tested in Section 2.2.

Following are the revisions:

“CDDP-TOS NPs (204.27 μM for CDDP) were lyophilized for ^1^H-NMR (400 MHz, DMSO-*d_6_*, Avance III HD, Bruker Corporation, Germany) and FT-IR (Nicolet 6700, American Thermal Power Company) testing to confirm the structure. The particle size and zeta potential of CDDP-TOS NPs (204.27 μM for CDDP) were measured ……”

8. *Check verb tenses throughout manuscript*

***Response:*** Thanks for your constructive suggestion. We have carefully checked the whole manuscript and made corresponding revisions (e.g., Line 27 in Section 1, line 6 in Section 2.6, line 16 and line 20 in Section 3.5 etc.), all of which have been highlighted in the revised manuscript.

9. *Specify the area of the plate for cell seeding in sections 2.7 and 2.8*

***Response:*** Thanks for your constructive advice. We have specified the area of the plate for cell seeding in Sections 2.6, 2.7, 2.8 and 2.9.

Following are the revisions:

“……. A549 cells were seeded in glass-bottom plates (d=35 mm, 2×10^5^ cells per well) and cultured for 24 h.

In brief, A549 cells were seeded in 6-well plates (d=35 mm, 2×10^5^ cells per well) and then treatment procedures were the same as the aforementioned CLSM observation.

For the flow cytometry tests, A549 cells were seeded in 6-well plates (d=35 mm, 2×10^5^ cells per well) and the treatment …….

A549 cells were seeded in glass-bottom plates (d=35mm, 2×10^5^ cells per well) and cultured for 24 h.”

10. *Include the concentration of particles used for DLS in figure 2*

***Response:*** Thanks for your constructive suggestion. We have added the concentration of TOS NPs and CDDP-TOS NPs in Figure 2.

Following are the revisions:

“Figure 2. ……. The size (B) and zeta potential (C) of TOS NPs (1.029 mM) and CDDP-TOS NPs (204.27 μM for CDDP). All data are expressed as mean ±SD (n=3).”

11. *Revise that sentences do not start with And*

***Response:*** Thanks for your suggestion. We have revised the sentences stared with And, including Section 2.2 line 19 and Acknowledgement line 3.

Following are the revisions:

“Then, the solution was dialyzed against PBS (pH=7.4) overnight and the FITC-labeled CDDP-TOS NPs were obtained.

…… (Grant No. 51973135). We would like to thank the Analytical & Testing Center of Sichuan University…….”

**References:**

1.	Zhang JW, Liu JL, Zhao YL *et al.* Plasma and cellular pharmacokinetic considerations for the development and optimization of antitumor block copolymer micelles. *Expert Opinion on Drug Delivery* 2015;12:263-281.

2.	Chou T-C. Theoretical basis, experimental design, and computerized simulation of synergism and antagonism in drug combination studies. *Pharmacological Reviews* 2006;58:621-681.
